# Clinical Instruction and Examination in Edinburgh

**Published:** 1907-08-17

**Authors:** 


// The Hospital

A Journal of

The Medical Sciences and Hospital Administration.

New Series. No. 21, Vol. I. [No. 1093, Vol. XLII.] SATURDAY, AUGUST 17, 1907.

### Clinical Instruction and Examination in Edinburgh.

A regulation recently adopted by the University of Edinburgh is likely to have important effects on medical teaching and education in Scotland. The regulation in question deals solely with the teaching of clinical surgery, but it is difficult to see how the principle it encloses can be restrained within so narrow a compass. There is reason, therefore, for careful scrutiny, as the new position may sooner or later affect the entire scope of medical education in the Scottish schools. The state of matters in relation to instruction in clinical surgery in Edinburgh has hitherto been as follows. The University possesses a Professor of Surgery and also a Professor of Clinical Surgery, and the holder of each of these offices is also a surgeon to the Royal Infirmary. In this institution he gives official clinical instruction to what is both in fact and name a formal University class that qualifies for graduation. At the same time, the Infirmary staff includes four other surgeons, and, upon application to the University Court, any one (or all) of these non-professorial surgeons may be "recognised" as a clinical teacher, and in these circumstances a certificate of attendance on his class will be accepted by the University for purposes of graduation. As a matter of fact, all these surgeons are "recognised" teachers in clinical surgery. They are not, however, within the organisation of the University, and they take no part in the University examinations. Here, indeed, is the essential difference between the surgeons who happen to be University professors and their colleagues. While all are clinical teachers, it is only the former who are examiners. Hence, almost inevitably, the classes conducted by the professors possess special attractions for the students, and those conducted by the other surgeons are thus placed at a disadvantage.

The proposal now adopted by the University Court will make all the surgeons to the Infirmary University Lecturers in Clinical Surgery. As a consequence, their classes, instead of being merely "recognised," will be part and parcel of the University scheme of teaching, equally with the classes conducted by the professors. It does not seem to be definitely stated, but the presumption is, that each of the lecturers, being now a University teacher, will take an appropriate part in the final examinations. In short, the University, instead of having, as hitherto, two *ex officio* teachers and examiners in clinical surgery, will have six such teachers and examiners. Thus, all the classes conducted at the hospital in this department will stand upon an equal footing, and the student, uninfluenced by extraneous considerations, will be free to select the teacher whose instruction appeals to his individual requirements. Such an arrangement must, of course, be grateful to the individual surgeons concerned, though possibly some of the conditions attached to it may be not altogether welcome. Of chief importance, however, is the question whether the new departure will conduce to the efficiency of the medical school, and to the successful instruction of the students. The answer to this question must, in our judgment, be decidedly in the affirmative. Clinical instruction, whatever else it may be, must be essentially practical, and must bring the student individually into contact with clinical facts. Large clinical classes cannot possibly satisfy this condition, and they inevitably mean for a large proportion of the students verbal and theoretical, instead of practical and personal instruction. So long as human nature is what it is, a teacher who is also an examiner will seem to the student a more desirable instructor to follow than his colleague who teaches but does not examine. The new plan, by putting all the teachers on one and the same footing, will thus help to secure a more equal distribution of the students. It will therefore tend to prevent the large and unwieldy classes which have heretofore existed, and in this way will so far secure the conditions which are essential to successful clinical teaching. We believe also that an increase in the number of examiners is a movement in the right direction. In a large school like Edinburgh, the final examinations must constitute an enormous strain on those whose duty it is to conduct them. With so wearisome and monotonous a task it must be difficult, indeed, to maintain both thoroughness of method and impartiality of judgment. To divide these duties will, we are confident, be in the interests both of the individual candidate and of the conscience of the examiner, while, by the agency of a board of studies and by appropriate grouping of the examiners, the maintenance of a uniform standard of examination may be readily secured. We believe, in short, that by the new departure the Edinburgh medical school has taken a step which will increase both its efficiency and its success.

The question will now certainly be asked, Why restrict the new arrangement to clinical surgery? Clinical medicine is in an identical position, and the claims of the one cannot be logically separated from the demands of the other. Further, if the new plan is good for Edinburgh, how can the other Scottish universities escape its adoption? All that can be said in reply is, that in practical affairs in this country logic is not the controlling influence which philosophers tell us it ought to be. A start has been made, and those who believe it to be in the right direction may wait with confidence for its results. Even if theoretical logic receives but little heed, the logic of the "stricken field" is inevitable. What has now been adopted partially and tentatively contains a living principle, and the operation of this over the entire field of Scottish medical education can therefore hardly be restrained.

